# Complete or High-Quality Draft Genome Sequences of Six Xanthomonas hortorum Strains Sequenced with Short- and Long-Read Technologies

**DOI:** 10.1128/MRA.00828-20

**Published:** 2020-10-08

**Authors:** Nay C. Dia, Fabio Rezzonico, Theo H. M. Smits, Joël F. Pothier

**Affiliations:** a Environmental Genomics and Systems Biology Research Group, Institute for Natural Resource Sciences, Zurich University of Applied Sciences (ZHAW), Wädenswil, Switzerland; b Molecular Plant Breeding, Institute of Agricultural Sciences, ETH Zurich, Zurich, Switzerland; University of Arizona

## Abstract

We report the genome sequences of six Xanthomonas hortorum species-level clade members, *X. hortorum* pathovars taraxaci, pelargonii, cynarae, and gardneri (complete genome sequences) and *X. hortorum* pathovars carotae and vitians (high-quality draft genome sequences). Both short- and long-read sequencing technologies were used.

## ANNOUNCEMENT

The genetic relatedness of the Xanthomonas hortorum species-level clade (slc) was established by Parkinson et al. using partial *gyrB* gene sequences (1). The most recently updated taxonomy of the clade (2) includes seven pathovars of *X. hortorum* (cynarae, gardneri, carotae, hederae, pelargonii, taraxaci, and vitians type B) (1–3).

We report here the whole-genome sequences of six *X. hortorum* strains (Table 1). The strains were isolated between 1942 and 2008 from a wide range of hosts and in various countries (Table 1). The genome sequences published in this work are either complete genome sequences or high-quality draft genome sequences, and all have five contigs or less (Table 1).

**TABLE 1:** Genome metrics and accession numbers of the newly sequenced genomes within the Xanthomonas hortorum species-level clade^*a*^

Strain^*b*^	Bacterial species	Origin (yr)	Host	Genome size (bp)	G+C content (%)	Total no. of genes	Genome status (*N*_50_ [bp])	No. of contigs/no. of plasmids	Illumina data:				Oxford Nanopore data:				SRA accession no. (MiSeq/MinION)	ENA accession no.
									I7/I5 indexes	Total no. of reads	Avg read length (bp)	Avg coverage (×)	BC	Total no. of reads	Read length *N*_50_ (bp)	Avg coverage (×)		
CFBP 498	*X. hortorum* pv. vitians	USA (1949)	*Lactuca* sp.	5,678,543	63.23	4,976	HQ draft (5,365,193)	4/3	N703/S504	1,112,420	259	38	BC18	27,882	9,898	58	ERR4326259, ERR4327117	LR828257 (chr.), LR828258 (p224), LR828259 (p47), LR828260 (p41)
CFBP 2044	*X. hortorum* pv. cynarae	France (1981)	*Cynara scolymus* L.	5,119,234	63.67	4,322	Complete	2/1	N705/S517	2,593,908	277	96	BC16	18,849	12,128	55	ERR4326260, ERR4327118	LR828251 (chr.), LR828252 (p40)
CFBP 2533^P^	*X. hortorum* pv. pelargonii	New Zealand (1974)	*Pelargonium peltatum*	5,287,542	63.72	4,427	Complete	3/2	N702/S517	3,398,636	261	111	BC15	14,695	8,396	20	ERR4326261, ERR4327119	LR828261 (chr.), LR828262 (p47), LR828263 (p41)
CFBP 7900^P^	*X. hortorum* pv. carotae	USA (2008)	*Daucus carota* (seed)	5,149,201	63.77	4,274	HQ draft (2,659,169)	5/ND	N702/S504	1,721,852	264	65	BC14	7,777	8,728	15	ERR4326262, ERR4327120	CAJDKC000000000
CFBP 8129	*X. hortorum* pv. gardneri	Costa Rica (1991)	*Solanum lycopersicum*	5,440,942	63.48	4,655	Complete	4/3	N705/S504	1,653,590	258	27	BC20	45,436	8,073	80	ERR4326263, ERR4327121	LR828253 (chr.), LR828254 (p211), LR828255 (p46), LR828256 (p26)
NCPPB 940^P^	*X. hortorum* pv. taraxaci	USA (1942)	*Taraxacum kok-saghyz*	5,029,134	63.83	4,316	Complete	2/1	N701/S503	1,197,722	269	43	BC17	9,847	7,654	18	ERR4329153, ERR4327122	LR828264 (chr.), LR828265 (p30)

a BC, barcode; HQ, high-quality; ND, not determined; chr., chromosome.

b The culture collections providing strains are abbreviated in the strain names as CFBP (Collection Française de Bactéries Associées aux Plantes, Beaucouzé, France) and NCPPB (National Collection of Plant Pathogenic Bacteria, York, United Kingdom). Superscript P following a strain name indicates the pathotype strain for the pathovar.

The strains were initially obtained as freeze-dried cultures in glass ampoules from two international strain collections abbreviated in the strain names as CFBP (Collection Française de Bactéries Associées aux Plantes, Beaucouzé, France) and NCPPB (National Collection of Plant Pathogenic Bacteria, York, United Kingdom). After revival on nutrient yeast extract glycerol agar plates (4) for 2 days at 28°C, an isolated colony was streaked onto the same medium and grown in the same manner. The strains were then stored in a −80°C ultrafreezer as glycerol stocks in 50% (vol/vol) nutrient yeast extract glycerol broth (4) until further use. The strains were revived as described above, and a single colony was used as starting material for downstream experiments.

Genomic DNA (gDNA) for Illumina MiSeq short-read sequencing was extracted from cells grown overnight at 28°C in nutrient yeast extract glycerol broth (4) using the NucleoSpin tissue kit (Macherey-Nagel, Düren, Germany) according to the manufacturer’s protocol with the following specifications: elution buffer was heated at 70°C before use, and the gDNA was eluted with 60 μl of this buffer. The quality of the gDNA was checked using a fragment analyzer (Advanced Analytical Technologies, Inc., Ankeny, IA) and quantified using the Quant-iT PicoGreen double-stranded DNA (dsDNA) quantification assay (Thermo Fisher Scientific, Waltham, MA). Library preparation was done using the Nextera XT DNA library prep kit (Illumina, San Diego, CA) following the manufacturer’s instructions. Sequencing was performed on a MiSeq Illumina sequencer with 2 × 300-bp paired-end reads using a MiSeq reagent kit version 3 (Illumina) according to the manufacturer’s instructions.

Genomic DNA for long-read sequencing was extracted from overnight-grown cells using the Gentra PureGene Yeast/Bact kit protocol (Qiagen, Hilden, Germany). The gDNA was quantified and its quality checked as described above. Library preparation and sequencing were performed with the ligation sequencing kit (catalog no. SQK-LSK109; Oxford Nanopore Technologies, Oxford, United Kingdom) and run on an R9.4.1 flow cell with a MinION sequencer. The native barcoding expansion kit (catalog no. XP-NBD114) was used for multiplexing. Base calling was performed using Guppy version 3.0.7 in the “accurate” mode implemented in the MinION release 19.06.8.

A hybrid assembly using the MiSeq and MinION reads was conducted with Unicycler version 0.4.7 (5). To check for misalignments, the MiSeq reads were mapped against the Unicycler assemblies using SeqMan Pro version 12.2 (DNAStar, Madison, WI). Contigs were reordered using the Mauve Contig Mover version 2.3.1 (6) when required (7). The scrub and align options of Unicycler were used to detect chimeras and check for high-error regions, respectively. The manual improvement of the genomes was finalized with Bandage version 0.8.1 (8). The genomes were then annotated using Prokka version 1.14.5 (9). Indels were checked using Ideel (10). All tools were run with default parameters unless otherwise specified.

The sizes of the hybrid assemblies ranged from 5,029,134 to 5,678,543 bp, a size range typically found in *Xanthomonas* genomes (Table 1). The G+C contents of the genomes varied from 63.23% to 63.83%, comparable to other *Xanthomonas* genome G+C contents. The sequenced genomes discussed here will be used for further analysis of evolution within the *X. hortorum* slc.

### Data availability.

The annotated genome sequences of the six *X. hortorum* strains have been deposited in ENA under BioProject no. PRJEB38812. The genome and raw read accession numbers for each isolate are shown in Table 1.
